# Evaluation of serum Amphiregulin levels in breast cancer patients and cancer-free controls

**DOI:** 10.1186/2162-3619-2-25

**Published:** 2013-09-08

**Authors:** Esther A Peterson, Eirini Pectasides, Shabana Shabbeer, Lisa Wiechmann, Joseph A Sparano, Paraic A Kenny

**Affiliations:** 1 Departments of Developmental & Molecular Biology, Albert Einstein College of Medicine, Bronx, NY 10461, USA; 2 Montefiore Medical Center, Bronx, NY 10461, USA; 3 Departments of Surgery, Albert Einstein College of Medicine, Bronx, NY 10461, USA; 4 Departments of Medicine, Albert Einstein College of Medicine, Bronx, NY 10461, USA; 5 Departments of Oncology, Albert Einstein College of Medicine, Bronx, NY 10461, USA

**Keywords:** Breast cancer, Serum biomarker, Amphiregulin, Epidermal growth factor receptor

## Abstract

**Background:**

Expression of the Epidermal Growth Factor Receptor ligand, Amphiregulin, has been associated with estrogen receptor positive breast cancer. As Amphiregulin is proteolytically released from the surface of breast cancer cells, we investigated the levels of Amphiregulin in the serum of breast cancer patients and cancer-free women to evaluate its potential utility as a breast cancer biomarker.

**Findings:**

Serum Amphiregulin levels were quantified by ELISA from 125 cancer-free women and 114 breast cancer patients. No significant association between serum Amphiregulin levels and breast cancer status was detected at two cut-points evaluated.

**Conclusions:**

Measurement of serum Amphiregulin levels lacks the necessary sensitivity and specificity for breast cancer screening in the general population.

## Background

Amphiregulin, a ligand of the epidermal growth factor receptor (EGFR), was initially identified as a secreted factor in the ERα-positive MCF7 breast cancer cell line [1], and was shown to be estrogen-responsive [2] in these cells. Our analyses of human breast tumors have demonstrated a strong correlation between ERα and Amphiregulin gene expression levels [3]. We have demonstrated that Amphiregulin is released from the cell surface by TACE/ADAM17 and drives EGFR pathway activation in several breast cancer cell lines suggesting that inhibiting the proteolytic production of EGFR ligands is a new mechanism by which to target EGFR signaling in cancer [3,4].

Assays such as PSA for prostate cancer and CA125 for ovarian cancer [5] have had a pronounced effect on the management of these diseases, but similarly useful markers in breast cancer are lacking. We recently reported the distribution of serum Amphiregulin in 85 cancer-free women [6]. In the current study, we have extended our cancer-free cohort to 125 samples and compare the distribution of serum Amphiregulin with two breast cancer cohorts: (1) women who had breast cancer surgery at least a year prior to enrollment and had no evidence of recurrent disease and (2) breast cancer patients with active disease.

## Methods

### Recruitment of human subjects

Female breast cancer patients ≥ 18 years, and receiving routine care at Montefiore Medical Center were recruited in two groups: (1) women who had breast cancer surgery at least a year prior to enrollment and no evidence of recurrent disease (n = 37) and (2) breast cancer patients with active disease (n = 77). For patients in the latter group with newly diagnosed disease (n = 44), Amphiregulin levels were measured prior to surgery, after removal of the tumor and at intervals thereafter. For the cancer-free cohort, serum samples were acquired from anonymous donors recruited by two commercial repositories (Innovative Research, Novi, MI and Promeddx, Norton, MA). Donors were non-pregnant women with no current or prior cancer diagnosis, and no history of diabetes, hepatitis B or C, or HIV. The study was approved by the Institutional Review Board of the Albert Einstein College of Medicine.

### Amphiregulin analysis

The human Amphiregulin DuoSet ELISA Development System (R & D Systems, Minneapolis, MN) was used to analyze Amphiregulin levels according to the manufacturer’s instructions. Although preliminary experiments suggested that Amphiregulin is detected similarly in serum and plasma, we tested our study hypothesis using only serum samples in order to minimize the potential contribution of technical variation. All measurements were made using the protocol we have previously described in detail [6]. All serum samples were stored at -80°C until analysis, and we found that measured Amphiregulin levels were stable over multiple freeze-thaw cycles. Sample concentrations were determined by interpolation using an eight-point standard curve, and samples exceeding the linear range of the assay (above approximately 1000 pg/ml) were diluted and analyzed again. Median Amphiregulin levels were compared between cohorts (Kruskal-Wallis test) and differences in the proportions of individuals with Amphiregulin levels above and below the evaluated cut-points (90th and 95th percentiles of the normal range) were tested using Fisher’s Exact Test.

## Results

**Figure 1:**
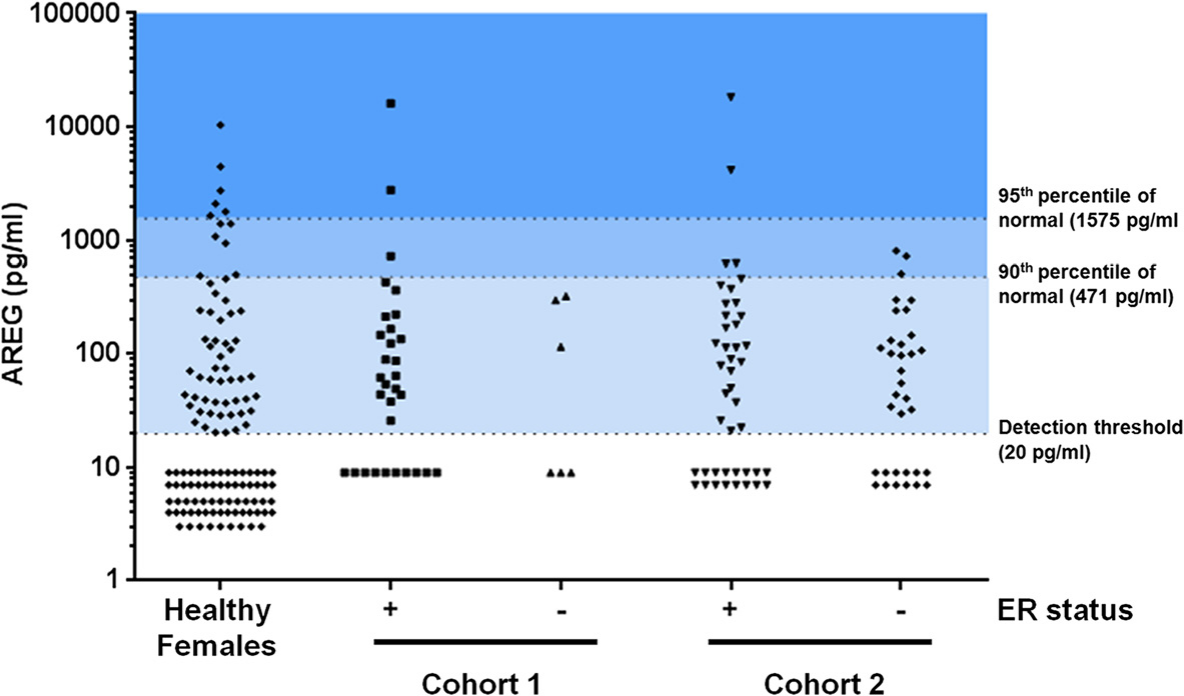
**Analysis of serum Amphiregulin levels in healthy females (n = 125), patients at least one year post-surgery with no evidence of recurrent disease (Cohort 1, 31 ER + and 6 ER-) and patients with active breast cancer (Cohort 2: 43 ER + and 34 ER-).** The 90th and 95th percentiles of the normal range are indicated by shading. The numbers of cases with no detectable serum Amphiregulin (<20 pg/ml) are indicated by points below the dashed line.

We previously reported the distribution of serum Amphiregulin in 85 cancer-free women, and demonstrated that these levels do not vary with stage of the menstrual cycle [6]. Here we have expanded this cancer-cancer free cohort to 125 women. The threshold of detection of the ELISA assay is 20 pg/ml and 69% of cancer-free women did not have detectable levels above this threshold. Women in the cancer-free cohort ranged in age from 18 to 78, and Amphiregulin levels were not correlated with age (R = 0.13). Median Amphiregulin levels were significantly higher than found in the cancer-free controls in the Cohort 1 ER + patients (P = 0.0068) and in the Cohort 2 ER + patients (P = 0.019) suggesting a shift towards Amphiregulin positivity in women with breast cancer. To determine if this shift was sufficiently strong to provide diagnostic utility, we evaluated two cut-points – the 90th percentile (471 pg/ml) and the 95th percentile (1575 pg/ml) of the normal range (Figure 1, Healthy Females).

We first evaluated the circulating Amphiregulin levels in women who had definitive breast cancer surgery at least one year prior to enrollment and in whom there was no evidence of recurrent disease (Figure 1, Cohort 1). These 37 women included 31 with prior ER-positive breast cancer and 6 women with prior ER-negative breast cancer. The proportion of these women with circulating Amphiregulin above either the 90th or the 95th percentile of the normal range was not statistically significantly different from the cancer-free population.

We then evaluated the circulating Amphiregulin levels in women with active breast cancer (43 with ER-positive and 34 with ER-negative breast cancer) (Figure 1, Cohort 2). In this cohort, there was also no difference in the distribution of serum Amphiregulin between either the ER-positive or ER-negative patients or between this cohort and the normal population.

**Figure 2:**
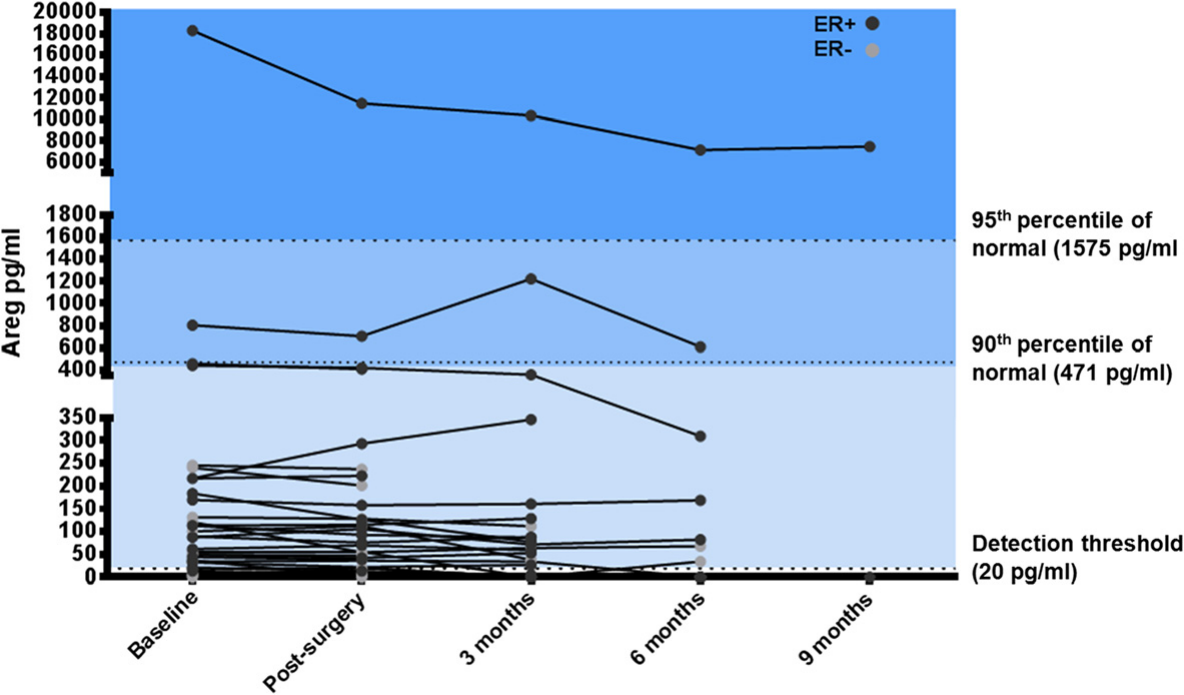
**Longitudinal analysis of serum Amphiregulin levels pre- and post-surgery and at three-monthly follow-up intervals.** This group of patients includes 30 ER + and 14 ER- patients.

We also performed longitudinal analysis of the patients in Cohort 2 that had been newly diagnosed with breast cancer prior to study enrollment. In these cases, serum samples were obtained before and after surgery, and at three monthly intervals thereafter. This subgroup included 30 patients with ER + breast cancer and 14 patients with ER-negative breast cancer. The majority of these patients had serum Amphiregulin levels within the normal range and these levels were not significantly altered following tumor removal (Figure 2). Notably, there was one outlier case with an extremely high baseline serum Amphiregulin level of 18,300 pg/ml. This level was reduced by 37% after surgery and continued to decline in follow-up analyses.

## Conclusions

Measurement of serum Amphiregulin levels lacks the necessary sensitivity and specificity for breast cancer screening in the general population.

## Consent

This study was approved by the Institutional Review Board of the Albert Einstein College of Medicine. Written informed consent was obtained from all patients for the publication of this report.

## Abbreviations

AREG: Amphiregulin; ELISA: Enzyme-linked immunosorbent assay; ER: Estrogen receptor.

## Competing interests

The authors declared that they have no competing interests.

## Authors’ contributions

PK and JAS conceived of the study. JAS, LW and EP enrolled patients in the study. EAP and SS quantified Amphiregulin in the samples. EAP and PK drafted the manuscript and all authors approved the final manuscript.
